# Supplementary material for: Reward signalling in brainstem nuclei under fluctuating blood glucose
Source: PLoS One. 2021 Apr 7;16(4):e0243899. doi: 10.1371/journal.pone.0243899 (PMC8026025; doi:10.1371/journal.pone.0243899)
Supplement: Attachment — Submitted filename: PlosOne reviewers comments _ Glucose reward paper .docx [file pone.0243899.s001.docx]

**Editors comments**

The main comment is the lack of methodological detail (see comments of both reviewers).

**We apologise for the inadequate detail. We have now substantially expanded and rewritten the methods section.**

It is impossible to currently understand how the TD model was constructed. Note that a TD model requires adding assumptions that may change its qualitative behavior (Pan et al., 2005, JNeurosci), so it must be made explicit.

**The relevant section now says:**

Equally important, I could not reconstruct how the 1st-level GLM was constructed (which events?...

**There were five events of interest, each of which had its own onset regressor: Cue_onset_ , Cue_high_ Cue_low_ Outcome_gluc_ Outcome_water_**

**...as detailed in the section copied above**

....which parametric modulators?...

**Each of these event onset regressors had 1st order parametric modulators obtained from interpolated values of serum glucose at the time of the event. This is now explained as follows:**

**All 1st order parametric modulators are orthogonal to their zero-th order modulators (the onsets), but no other orthogonalisation was performed. (stated above).**

...and how contrasts were subsequently defined based on the 1st-level regressors.

**Contrasts are now more explained in the section on modelling RPEs copied above.**

Relatedly, it seems that the modulation by serum glucose runs opposite to what the authors predicted (more glucose, stronger response). If so, this must be made explicit in the Discussion**.**

**Yes, this is important to rectify. The opening paragraph of the discussion now foregrounds this as follows:**

**Reviewer comments**

**Reviewer #1:** The authors propose a study aimed at investigating the influence of homeostatic state on RPE signals. They manipulated homeostatic state by varying the blood glucose levels with two paradigms, one ascending (start low and grow) and the other descending (start high and decrease). The results indicate a clear modulation RPE x glucose level in several cortical and subcortical areas, including the VTA, PBN, and caudate nucleus. The homeostatic modulation of RPE was found to depend also on the glucose level phase (ascending or descending). I found the study interesting and very relevant for the understanding of the biological/evolutionary meaning of RPE. I have no major concerns about the methods, nonetheless the authors should improve the clarity of results exposition, in order to make their findings more easily understandable by the reader. In particular (sorted by appearance order in the manuscript):

1. Figure 1b. The authors should define already here as “ascending” and “descending” the two glucose conditions. Moreover, it is not specified the meaning of the eight different time courses in each condition.

**Now explained. It now says:**

***The different line plots indicate different sessions for all subjects.***

2. Figure 2. I suppose the y axis indicates PSC. It should be specified.

**Correct. The end of the figure legend now says:**

***...in units of percent signal change.***

The authors should stress out the matching between the meaning of the time courses color here and in Figure 1d

**Yes, the legend now says:**

***The colours of time courses follow the same meaning as shown in Fig. 1c-d.***

3. Figure 3b and Figure 4b are hard to understand. I think the trajectory furthest away from the viewer does not provide important information, it is difficult to read and confounds the reader (it seems an additional trajectory besides those described in Figure 1d). Moreover, the authors should help the reader in matching these trajectories with those described in Figure 1d (who’s who?).

**The figure legends now read:**

**We understand the point about the fifth set of trajectories being confusing, however we would prefer to keep it in, since it affords comparison with Fig. 1d. Hopefully we are now clearer about it what all these trajectories mean in the legend.**

Finally, recent literature on computational neuroscience of Reinforcement Learning (RL) is evidencing how decision-making in the mammalian brain is strongly driven by optimizing the net value (discounted by costs) about both primary and non-primary rewards (e.g. Alexander & Brown 2011; Silvetti, Alexander et al. 2014; Verguts et al. 2015; Silvetti, Vassena et al., 2018). I think this work is relevant for clarifying how reward and RPE signals are dependent from internal states, and how the latter modulate RL processes, suggesting that RPEs are not only the comparison between expectation and environmental outcome (objective), but are dependent on homeostatic states (subjective). These results should be linked to the above literature in the Discussion section, as they contribute to a paradigmatic change that shifts RL-based decision-making from being “objective” to be more “subjective”.

**This is now discussed in the discussion as follows:**

***Recent literature on the computational neuroscience of reinforcement Learning (RL) has evidenced how decision-making in the mammalian brain is driven by optimizing the net value of both primary and non-primary rewards. Such reward computations have been shown to rest on a comparison between the expectation and outcome of external environmental cues, integrating both the physical and cognitive effort costs of the agent (e.g. Alexander & Brown 2011; Silvetti, Alexander et al. 2014; Verguts et al. 2015; Silvetti, Vassena et al., 2018). The work presented here tentatively expands this perspective by showing that reward and RPE signals are dependent on internal homeostatic states, which may serve to modify the motivational values according to the idiosyncratic and time varying homeostatic needs of the organism.***

Reviewer #2: Morville and colleagues use FMRI to investigate the relationship between reward prediction error (RPE) signals and homeostatic state (ascending and descending serum glucose trajectories). The authors find that RPE responses in midbrain structures are sensitive to glucose trajectories, which demonstrates a link between RPE responses and homeostatic processes. The authors acknowledge several important limitations in their work, and draw generally appropriate conclusions from their findings. Although I think the manuscript possesses many good features, I have a number of concerns that should be addressed in a revision.

Major Concerns

1) My biggest problem with the manuscript is the lack of methodological details. First, it is difficult to evaluate what was done with respect to the analyses. I think the authors rely on the canonical hemodynamic response function. But, it's not clear to me if this shape would capture meaningful variation tied to difference in neuronal activity in this design (cf. Chen et al., 2015). Some discussion of the potential impact of HDR shape would be important to include.

**The model included temporal and dispersion derivatives. This was not detailed in the methods. It now reads (underlined indicates new edits):**

***All variables of interest were convolved with the canonical hemodynamic response function, along with their associated temporal and dispersion derivatives function and fitted to the data using the specified GLM.***

**We searched extensively for Chen et al 2015, with various key words pertaining to hemodynamics and reward however we could not figure which paper is being referred to. In any case we now discuss the form of the HDR as follows:**

***Another issue is whether the hemodynamic modelling was appropriate for detected evoked responses in subcortical regions which may deviate from the canonical hemodynamic response functions typically used. The regression model we used deployed temporal and dispersion derivatives for all regressors of interest in order to account for idiosyncratic variance in the timing and temporal spread of the hemodynamic response function.***

Second, it is not clear how the authors modeled RPEs and included those responses as parametric modulators in their analyses.

**We try to explain this more clearly. We have largely rewritten and expanded the relevant sections, as follows:**

Third, given the design and concatenation of the FMRI sessions, how did this interact with the high pass filter?

**The modulations of glucose are on events that are happening at higher frequencies than those filtered out by the high pass filter. This is discussed as follows:**

2) The authors state that they will release the data on NeuroVault. However, this repository is for statistical maps, which should be shared of course (e.g., Botvinik-Nezer et al., 2020). I recommend that the authors share the raw data (formatted into the Brain Imaging Data Structure) on OpenNeuro (Gorgolewski et al., 2015). With such a rich dataset and many important open questions (e.g., fitting the RL model directly to the brain data), it would be unfortunate not to share the data openly and publicly. (Of course, I realize the authors might still be working on some of these questions, and thus could elect to embargo the data for some period of time. But, as is, the manuscript is not compliant with data sharing policies at this journal and hence why I list this as a "major" comment.)

**We advocate for the open sharing of data, and all our present projects from 2019 onwards are shared openly and encoded in BIDS format. The problem with doing this for this dataset from 2016 is that translating into BIDS retrospectively takes a lot of time (a recent project took several months to completely encode as BIDS) and it means none of the analysis scripts will work any more. At present, due to resource constraints (no funding, and since the student has moved to industry) we cannot at this time agree to upload to OpenNeuro. At the time of stating our data sharing policy we actually didn’t realise Neurovault was for statistical maps only. Instead we propose to upload the full dataset and analysis scripts to the open science framework, using practices in compliance with PLOS One. We hope this will not preclude publication.**

**We have no need for embargo.**

Minor Concerns

1) Although the paper from Stauffer and colleagues (2014) links dopaminergic activity to marginal utility and the explanation in the Introduction makes sense to me, I think it would be worth explaining this concept further in the Discussion. Unless I missed it, the authors do not mention marginal utility again after the Introduction.

**We now return to it in the opening paragraph of the discussion, and in doing so make it clearer that our main hypotheses were actually not corroborated.**

2) I think the concept of "three interacting valuation systems" (Sternson & Eiselt 2017) could get confused with other valuation systems, as described in related work (e.g., Rangel et al., 2008). Please try to clarify and reconcile these different frameworks.

**Yes. Confusion is perhaps needlessly caused by the term valuation systems. By deleting the word valuation we hope that this confusion is avoided.**

3) The reward cue and liquid would be very difficult to separate without any jitter. In addition. the responses to these events could occur in the same or consecutive TRs. Please clarify how these phases of the task were modeled (see above major comment).

**They are decoupled statistically by the probabilistic transition between cue and outcome. We now state this as follows:**

***Whilst there is no jitter between the cue and the outcome, independent estimation of each of the effects is achievable by virtue of their probabilistic decoupling.***

4) How were the scanning parameters optimized to record midbrain responses? Were there any corrections for physiological noise?

**Yes, we now explicitly state this.**

**Correction for the physiological noise is already stated here, but we added the reference:**

5) How did the authors ensure that participants were compliant with the 12-hour fasting protocol?

**We now state:**

***Compliance with the 12 hour fasting was based on trust, and on the understanding that we would be able to detect if they had not fasted via blood tests. Any participant not in a hypoglycemic range at the start of the experiment would be assumed to be not having fasted and the session would be aborted. This was not necessary in any participant.***

6) Please check figure legends for completeness/accuracy. Some elements (e.g., line color for the HDR functions in Figure 2 are not defined).

**Line colour is defined now for Figure 2, other figure legends have been checked and edited.**

7) In Figure 2, please add error bars to the HDR functions.

**Because there are four overlapping trajectories, adding upper and lower bounded trajectories is visually confusing, however we now add error bars to the peak of each trajectory. Since this is a single summary statistic it represents the error on the whole trajectory.**

8) In Figures 3 and 4, consider using a different color bar for the brain activation and serum glucose levels. As is, the color bars looks somewhat similar, which could lead to confusion.

**Instead of remaking the two figures we now draw the readers attention to the fact that the color bars denote different meanings. The legend now reads:**

***Note that the colour bar for the graphs in (b) is serum glucose, and is distinct from the colour bar in (a) used to indicate t-values.***
