# Supplementary material for: Reward signalling in brainstem nuclei under fluctuating blood glucose
Source: PLoS One. 2021 Apr 7;16(4):e0243899. doi: 10.1371/journal.pone.0243899 (PMC8026025; doi:10.1371/journal.pone.0243899)
Supplement: Attachment — Submitted filename: Plos One reviewer comments rFlux.docx [file pone.0243899.s002.docx]

**Reviewer and editor comments PloS One Morville et al.**

The study design is not entirely clear: Every subject does 4 sessions, but is it LLHH, or LHHL, or a random order,…?

**Random order. We now state this on page 4:**

***The temporal order of the preload conditions was randomised within subjects, with each condition being performed twice.***

P 6: Ascending and descending are used for the first time here; and only explained on page 10. Please define on first pass.

**This is now explained when introducing the preloads on page 4:**

***The lo-glucose preload resulted in glucose ascending during the fMRI acquisition period, due to the consumption of glucose, whereas during the same period after the hi-glucose preload, glucose levels descended (Fig. 1b). The preload conditions are thus referred to as ascending and descending conditions.***

P 6: “A standard fixed effects level…” Do you mean at a fixed effects level model was fitted across all data? Although a fixed effects model in itself is standard, it is definitely not standard to apply it across subjects (without random effects). Please explicitly comment on this in the paper.

**We now remove the word standard. We also add the following statement in the discussion concerning fixed effects (italics):**

***Due to the small number of subjects, we deployed a fixed effects analysis over all subjects. It should be noted that this makes assumptions about the nature of the noise that might not be compatible with the repeated measures design, since it is difficult to correct for non-sphericity in this setting.* Future work will expand this paradigm with a larger group of subjects to afford random effects modelling, and thus generalisation to the population sampled from.**

The model clarification is good, but I wonder about the statement “Since there is no overt behaviour…” This is true, but i don’t see how this is relevant. Behavioral data are needed indeed when you want to estimate the parameters of a model; but you can perfectly well generate regressors for a Rescorla-Wagner, TD, or any other model (which, apparently, is what you do). TD is just about how you model how value “percolates” to earlier time points (such as cue) from reward. You can generate regressors for that model. But the way I see it, your model does not learn, but you give it for each time point (cue, feedback, …), the equilibrium value that it has to converge to, based on the statistics of the experiment? Then say explicitly that your model does not learn, not that you don’t have behavioral data to fit a model.

**We agree that the model does not learn and that this is important to point out. We now say on page 7:**

***It should be noted here that in this approach to modelling RPEs, the model does not incorporate any effect of learning, in effect modelling what signals are expected once learning has converged on the expected reward values.***

**There being no overt behavior is the main reason why we did not fit a learning model, because there is no behavior to fit the model to, as is typically done. Thus we think it is relevant to include as a statement, given what is likely expected by the reader for this kind of experiment. For that reason we prefer to keep the statement which says:**

***Since there is no overt behavior in this task to which a temporal difference learning algorithm can be fit, we used a different approach based on how RPEs converge to changes in the conditional expectation values of reward outcomes.***

P 12: “that this was true”: change to “this was true”

**Done.**

**We think the paper has been much improved following the reviewers’ and editors comments. We hope that you find that these changes are satisfactory and we can now proceed with publication.**
